# Fatty infiltration of the multifidus muscle independently increases osteoporotic vertebral compression fracture risk

**DOI:** 10.1186/s12891-023-06640-2

**Published:** 2023-06-22

**Authors:** Dong Gyu Lee, Jae Hwa Bae

**Affiliations:** Department of Physical Medicine and Rehabilitation, College of Medicine, Yeungnam University, 317-1, Daemyungdong, Namku, Daegu, 705-717 Republic of Korea

**Keywords:** Osteoporotic vertebral compression fracture, Multifidus muscle, Fatty infiltration, Back muscle atrophy, Bone mineral density

## Abstract

**Background:**

Vertebral compression fractures decrease daily life activities and increase economic and social burdens. Aging decreases bone mineral density (BMD), which increases the incidence of osteoporotic vertebral compression fractures (OVCFs). However, factors other than BMD can affect OVCFs. Sarcopenia has been a noticeable factor in the aging health problem. Sarcopenia, which involves a decrease in the quality of the back muscles, influences OVCFs. Therefore, this study aimed to evaluate the influence of the quality of the multifidus muscle on OVCFs.

**Methods:**

We retrospectively studied patients aged 60 years and older who underwent concomitant lumbar MRI and BMD in the university hospital database, with no history of structurally affecting the lumbar spine. We first divided the recruited people into a control group and a fracture group according to the presence or absence of OVCFs, and further divided the fracture group into an osteoporosis BMD group and an osteopenia BMD group based on the BMD T-score of -2.5. Using images of lumbar spine MRI, the cross-sectional area and percentage of muscle fiber (PMF) of the multifidus muscle were obtained.

**Results:**

We included 120 patients who had visited the university hospital, with 45 participants in the control group and 75 in the fracture group (osteopenia BMD: 41, osteoporosis BMD: 34). Age, BMD, and the psoas index significantly differed between the control and fracture groups. The mean cross-sectional area (CSA) of multifidus muscles measured at L4-5 and L5-S1, respectively, did not differ among the control, P-BMD, and O-BMD groups. On the other hand, the PMF measured at L4-5 and L5-S1 showed a significant difference among the three groups, and the value of the fracture group was lower than that of the control group. Logistic regression analysis showed that the PMF value, not the CSA, of the multifidus muscle at L4-5 and L5-S1 affected the risk of OVCFs, with and without adjusting for other significant factors.

**Conclusions:**

High percentage of fatty infiltration of the multifidus muscle increases the spinal fracture risk. Therefore, preserving the quality of the spinal muscle and bone density is essential for preventing OVCFs.

## Background

Vertebral compression fractures decrease daily life activities and increase economic and social burdens [1]. In the United States, it is reported that 700,000 patients experience osteoporotic vertebral compression fractures (OVCFs) each year, and about 40% of women experience OVCFs in their lifetimes [2]. Osteoporotic fractures, including vertebral compression fractures, occur when bone strength is compromised by trauma ranging from normal lifting and bending to high-impact falls [3].

According to the literature, as age progresses, the bone mineral density (BMD) and bone quality of the spine decreases, which increases the incidence of OVCFs [4–7]. OVCFs not only cause acute or chronic pain but also cause structural abnormalities, such as thoracic kyphosis or lumbar lordosis, resulting in reduced exercise tolerance and thoracic space. These influences lead to emotional problems, such as a decrease in self-esteem [8], physical limitations, and ultimately a lower quality of life (QoL) in older people [9]. Spinal osteoporosis itself also reduces the spinal range of motion and movement velocity, which causes functional impairment, which in turn reduces the QoL [10]. Therefore, early diagnosis and treatment of spinal osteoporosis in older people can decrease the risk of OVCFs [11].

Clinically, spinal BMD measurement is the principal diagnostic tool for spinal osteoporosis. A T-score for BMD < -2.5 has been regarded as the diagnostic criterion for osteoporosis. A decreased BMD increases the risk of an OVCF [4]. However, OVCFs can still occur even when the T-score for BMD is over − 2.5. Factors other than BMD, such as age, smoking status, alcohol intake, and body composition, can also affect spinal compression fractures [12, 13].

Sarcopenia has been a noticeable factor in the aging health problem [14]. According to the definition of sarcopenia revised in 2018 by the European Working Group on Sarcopenia in Older People, it usually refers to low muscle strength but is confirmed when there is accompanying low muscle mass or quality [15].

Currently, researchers are paying attention to the interconnection and bidirectional influence of muscle and bone metabolism [16]. In sarcopenia, muscle strain decreases, suggesting that bone metabolism through osteocytes will decrease. Moreover, the multifidus muscle stabilizes the segmental spine to prepare for movement. In sarcopenia, dynamic stabilization is not achieved when the segmental spine is loaded due to atrophy of the multifidus muscle, so a force can be focused on the focal area of the segmental spine or a single level of the spine [17, 18]. These localized forces compromise bone strength, which can increase the vulnerability to OVCFs [3].

Therefore, we hypothesized that the quantity and quality of the multifidus muscle would decrease as sarcopenia develops with age, thereby increasing the incidence of OVCFs. We aimed to evaluate the extent to which the quality and quantity of the multifidus muscle affect OVCFs in individuals aged > 60 years.

## Methods

### Study population

The study population was retrospectively recruited from patients who visited the university hospital between January, 2020 and April, 2022. The institutional review board (YUMC 2022-08-045) approved this study and waived the requirement for informed consent. Among the recruited patients, participants were selected according to the following criteria. The inclusion criteria were: (1) age ≥ 60 years (2) available cross-section scan images of the lumbar spine on lumbar spine magnetic resonance imaging (MRI), and (3) concurrent BMD and lumbar spine MRI. The exclusion criteria were: (1) history of lumbar spinal surgery, (2) cancer, (3) spine infection, (4) severe degenerative scoliosis, and (5) systemic diseases affecting bone density, including chronic renal failure and liver cirrhosis.

The sample size was calculated using a power of 0.8, effect size of 0.5, and allocation ratio of 0.5 by the G-power program. This calculation yielded a sample size of 114, with 76 and 38 in fracture group and control group, respectively.

A clinically skilled radiologist with more than 10 years of experience, specializing in neurology, head, and spine, diagnosed OVCF based on lumbar spine MRI images. The participants were divided into three groups based on lumbar spine BMD and OVCFs. The control group underwent BMD and spinal MRI evaluation but did not have OVCF. Based on the T-score of lumbar spine BMD [19], the fracture groups with OVCF were divided into the osteopenia and osteoporosis groups for subgroup analysis. The osteopenia BMD group (P-BMD) showed osteopenia T-scores of spinal BMD of over − 2.5. The osteoporosis BMD group (O-BMD) showed osteoporosis T-scores of lumbar BMD of -2.5 and below.

### Psoas and multifidus measurement using spine MRI

FIJI software (http://fiji.sc/Fiji) was used for quantitative analysis. Semiautomated methods using FIJI software measured the cross-sectional area (CSA) of the psoas and multifidus muscles in the same manner as in a previous study [20].

**Fig. 1:**
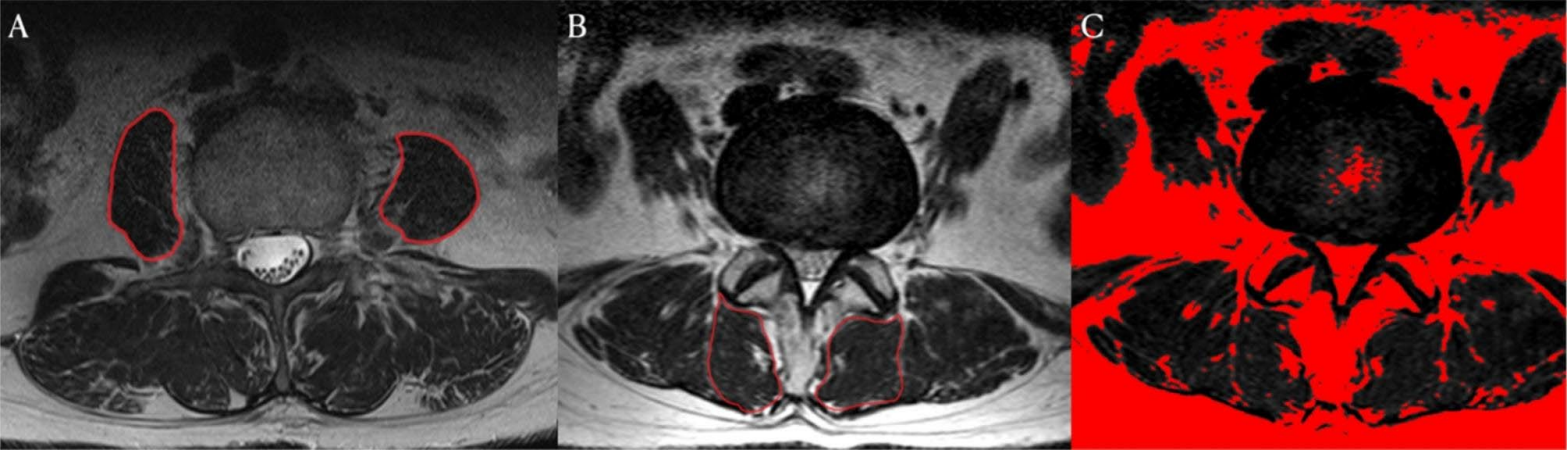
Psoas and multifidus measurement using spine MRI. The psoas and multifidus muscles were drawn directly on axial T2-weighted magnetic resonance (MR) images. (A) The psoas muscle’s cross-sectional area (CSA) was manually selected at the upper level of the L3 vertebral body. (B) CSA of the multifidus muscle is marked at L4–5 and L5–S1 levels, respectively. (C) The fat area of muscles on the T2-weighted MR image was converted to red using the pseudo-coloring technique in the software based on the threshold. The percentage of muscle fibers (PMF) in the multifidus muscle was calculated by subtracting the red area from the CSA and multiplying it by 100

The CSA of the psoas muscle was manually measured at the L3 vertebral body level using T2-weight axial spinal MRI (Fig. 1. A). The psoas index (cm^2^/m^2^) was calculated by dividing the CSA (cm^2^) of both sides by height squared (m^2^) [21].

The CSA of the multifidus muscle at L4-5 and L5-S1 was manually measured using T2-weight axial spinal MRI (Fig. 1. B). The fat area of the multifidus muscle at L4-5 and L5-S1 was determined using the threshold analysis (Fig. 1. C). The optimal threshold for fat tissue was manually determined [22]. The percentage of muscle fibers (PMF) in the multifidus muscle at L4-5 and L5-S1 was calculated by subtracting the fat area from the CSA and multiplying it by 100.

### Statistical analyses

Statistical analysis was performed using the Statistical Package for Social Sciences (version 22.0; SPSS Inc., Chicago, IL, USA). If the continuous variable followed a normal distribution, it was expressed as mean ± standard deviation, and if it did not follow a normal distribution, it was expressed as median (lower quartile, upper quartile). Variants between the control and fracture groups were analyzed using a t-test or Mann Whiteny U test. Analysis of variance (ANOVA) was used to evaluate whether there were differences in age, body mass index (BMI), bone density, and muscle components among the groups (control, P-BMD, and O-BMD). A post hoc test was conducted to analyze significant differences among the groups. The PMF of the fracture and lower lumbar levels were evaluated using ANOVA to determine whether there was a significant difference. To evaluate the effect of CSA and PMF on OVCFs, we used logistic regression analysis. We used the presence or absence of OVCFs as the objective variable, and the CSA and PMF of the multifidus muscle at two lower lumbar levels (L4–5, L5–S1) as explanatory variables. Additionally, logistic regression analysis was performed by adjusting for age, BMI, BMD, and psoas index. The Hosmer–Lemeshow goodness-of-fit test was performed to test the goodness-of-fit of the logistic regression model for each variable. Statistical significance was set at p < 0.05.

## Results

We retrospectively included 120 patients who had visited a tertiary referral hospital. Based on spine MRI, 75 participants were diagnosed with OVCFs. Of the 75 participants with OVCFs, 41 were in the P-BMD group, and 34 were in the O-BMD group (Table 1). In the control group, the age range was 60 to 81 years, and in the OVCF groups, was 61 to 92 years. In the OVCF groups, the age range of the O-BMD group was 62 to 92 years, and of the P-BMD group was 61 to 89 years. In total, 12, 3, and 0 patients had a normal BMD range (T-score >-1) in the control, P-BMD, and O-BMD groups, respectively.

**Table 1:** Demographic data and analysis of variance according to the groups

		Total (n = 120)	Control group (n = 45)	Fracture group		P value
				P-BMD (n = 41)	O-BMD (n = 34)	
Age (year) M’(Q_1_, Q_3_)		72(68, 79)	69(66, 73)	75(69, 80)		< 0.05*
				78.00(70, 82)	71.00(68, 76)	< 0.05*
BMI (kg/m^2^) M ± SD		23.49 ± 3.42	24.53 ± 3.44	22.87 ± 3.28		0.09
				24.02 ± 2.94	21.47 ± 3.16	< 0.05*
Spinal T-score M ± SD		-1.73 ± 1.40	-0.85 ± 1.21	-2.25 ± 1.23		< 0.05*
				-1.40 ± 0.83	-3.29 ± 0.72	< 0.05*
Psoas index (cm^2^/m^2^) M ± SD		4.91 ± 1.21	5.21 ± 1.13	4.72 ± 1.23		< 0.05*
				4.93 ± 1.25	4.48 ± 1.17	< 0.05*
Multifidus on L4-5	CSA (cm 2) M’(Q_1_,Q_3_)	1214.11 (1060.53, 1383.06)	1180.22(959.11,1379.50)	1278.81(1100.16,1433.57)		0.23
				1211.76(1072.46,1381.25)	1294.78(1122.88,1459.52)	0.40
	PMF (%) M ± SD	61.69 ± 17.24	74.89 ± 10.20	53.78 ± 15.71		< 0.05*
				47.06 ± 13.25	45.20 ± 18.40	< 0.05*
Multifidus on L5-S1	CSA (cm 2) M ± SD	1351.95 ± 358.18	1311.09 ± 341.07	1376.47 ± 368.13		0.33
				1400.47 ± 337.55	1347.52 ± 405.22	0.51
	PMF (%) M ± SD	61.85 ± 14.39	70.12 ± 12.36	56.89 ± 13.25		< 0.05*
				55.12 ± 12.70	59.03 ± 13.77	< 0.05*
Fracture Level (number of patients)	T10	2	0	2	0	
	T11	6	0	4	2	
	T12	14	0	7	7	
	L1	23	0	13	10	
	L2	8	0	2	6	
	L3	8	0	6	2	
	L4	9	0	4	5	
	L5	5	0	3	2	

The significance level was set at P-value < 0.05. * Significant differences were determined using analysis of variance. PMF: Percentage of muscle fibers in the multifidus muscle. M: Mean, SD: Standard deviation, M’: Median, Q_1_ : Lower quartile, Q_3_ : Upper quartile

In Table 1, age, spinal T-score, and psoas index differed significantly between the control and fracture groups (p < 0.05). There were also significant differences when comparing the control, P-BMD, and O-BMD groups (p < 0.05). BMI differed significantly among the control, P-BMD, and O-BMD groups (p < 0.05), but not between the control and fracture groups (p = 0.09).

While the CSA of the multifidus muscle did not show a significant difference, the PMF showed a significant difference between the control and fracture groups or among the control, P-BMD, and O-BMD groups, at the L4–5 and L5–S1 levels, respectively (p < 0.05). In addition, the mean CSA of the multifidus muscles on L4–5 and L5–S1 was higher in the fracture group (1274.00 and 1376.47, respectively) than in the control group (1180.22 and 1311.09, respectively). The mean PMF of the multifidus muscles on L4–5 and L5–S1 was lower in the fracture group (53.78 and 56.89, respectively) than in the control group (74.89 and 70.12, respectively).

**Fig. 2:**
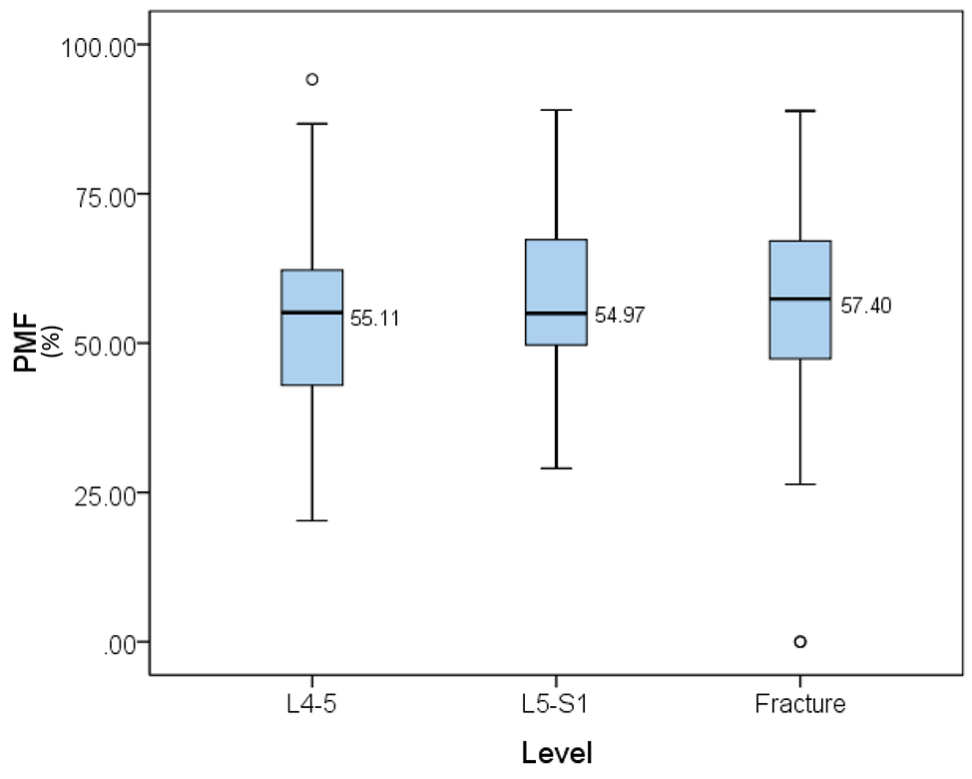
PMF measured at L4–5, L5–S1, and disc level with fracture. There was no significant difference between each distribution of PMF measured at L4–5, L5–S1, and disc level with fracture. PMF, percentage of muscle fibers in the multifidus muscle

As seen in Table 2, the PMF of the multifidus muscle at L4–5 and L5–S1, but not the CSA of the multifidus muscle, significantly affected the risk of OVCFs. The high PMF value of the multifidus muscle at L4-5 significantly lowers the risk of OVCFs by 12% when other factors are not adjusted (crude odds ratio [COR] = 0.88; 95% confidence interval [CI] 0.84–0.92, p-value < 0.05), whereas 13% even when age, BMI, BMD, and psoas index are adjusted (adjusted odd ratios [AOR] = 0.87; 95% CI 0.82–0.92, p-value < 0.05). The high PMF value of the multifidus muscle at L5-S1 significantly lowers the risk of OVCFs by 8% when other factors are not adjusted ([COR] = 0.92; 95% CI 0.89–0.95, p-value < 0.05), and 9% even when age, BMI, BMD, and psoas index are adjusted ([AOR] = 0.91; 95% CI 0.88–0.95, p-value < 0.05). On the other hand, the low CSA value of the multifidus muscle at L4-5 or L5-S1 did not affect the risk of OVCFs regardless of whether the factors were adjusted or not(the CSA of the multifidus muscle at L4–5: [COR] = 1.00; 95% confidence interval [CI] 1.00–1.00, p-value = 0.25, [AOR] = 1.00; 95% CI 0.99–1.00, p-value = 0.38; the PMF at the multifidus muscle at L5–S1: [COR] = 1.00; 95% CI 0.99–1.00, p-value = 0.33, [AOR] = 1.00; 95% CI 1.00–1.00, p-value = 0.10). There was no significant difference between each distribution of PMF measured at L4–5, L5–S1, and the disc level with the fracture (Fig. 2).

**Table 2:** Multivariate logistic regression analysis for the osteoporotic compression fracture risk according to quantity and quality of multifidus muscles

Variables		Fracture Risk			
		Crude		Adjusted^a^	
		OR (95% CI)	P value	OR (95% CI)	P value
CSA of multifidus	on L4-5	1.00 (1.00, 1.00)†	0.25	1.00 (0.99, 1.00)†	0.38
	on L5-S1	1.00 (0.99, 1.00)†	0.33	1.00 (1.00, 1.00)†	0.10
PMF of multifidus	on L4-5	0.88 (0.84, 0.92)	< 0.05*	0.87 (0.82, 0.92)†	< 0.05*
	on L5-S1	0.92 (0.89, 0.95)†	< 0.05*	0.91 (0.88, 0.95)†	< 0.05*

OR, odds ratio; CI, confidence interval; CSA, cross-sectional area; PMF, percentage of muscle fibers in the multifidus muscle calculated by subtracting the fat area from the CSA and multiplying it by 100

* P < 0.05

† P value of Hosmer and Lemeshow goodness-of-fit tests > 0.05

^a^ Adjusted for age, body mass index, bone mineral density, and psoas index

## Discussion

In this study, we measured CSA and PMF of multifidus muscle as indicators of muscle quantity and quality, and evaluated whether they affect the incidence of OVCFs. As shown in Table 2, the CSA, which represents the quantity of the multifidus muscle, and the PMF, which represents the quality of the multifidus muscle, showed different results. The CSA of the multifidus muscle did not show a relationship with the incidence of OVCFs. However, the PMF of the multifidus muscle significantly affected the incidence of OVCFs. These significant associations appeared even after adjusting for previously investigated confounding factors. Furthermore, the high percentage of fatty infiltration of the multifidus muscle increased the risk of spinal fracture, even though the T-score of BMD indicated osteopenia. These findings suggest that preserving spinal muscle quality, regardless of BMD, is important in preventing OVCFs.

Clinically, BMD evaluation provides a guideline for osteopenia and osteoporosis diagnoses [23, 24] A T-score of BMD < -2.5 has been regarded as the diagnostic criterion for osteoporosis. However, the T-score of the BMD was not a decisive factor for spinal fractures. Sometimes, there was compression fracture in patients with a T-score higher than − 2.5 in BMD. According to a previous study, the diagnosis of osteoporosis based solely on T-score did not align well with fragility fractures, as indicated by low Cohen and Younden indexes (< 0.4) [25]. Therefore, while a T-score of BMD below − 2.5 increase the probability of fractures, it’s essential to consider the interactions with other risk factors that can further contribute to an increased fracture probability [25, 26].

Previous studies have shown that age, BMD, sarcopenia, and BMI increase the risk of OVCFs, which is consistent with our findings [12, 27, 28]. Among these, sarcopenia has received attention as a potential factor influencing osteoporotic fracture, with its increase in the aging population [29]. Therefore, we tried to elucidate the effects of sarcopenia-induced decrease in back muscle quantity and/or quality on spinal compression fractures.

The multifidus muscle enhances the stabilization of the spine [30]. They have a short extending length, so their fibers are packed densely within a small volume. The high stiffness of their fibers increases the resistance of lumbar spine flexion. Thus, the lumbar back muscle atrophy increases compression and shear force within the disc level [31]. The patients with OVCFs showed a significant increase in spinal flexion load than those without OVCF [32]. Consequently, multifidus muscle atrophy increases the flexion force and decreases the spinal segment stability, resulting in an increased risk of OVCFs.

In this study, we measured the CSA along the cortical margin of the multifidus muscle on MRI. This is an indicator that can confirm the degree of atrophy of the entire multifidus muscle which is composed of muscle fibers and internal fatty infiltration. However, the CSA of the multifidus muscle did not show significant differences among the groups, not even between the fracture and control groups. On the other side, PMF of the multifidus muscle significantly affected the OVCF.

As atrophy of the deep back muscles is generally present in older patients, preserving functioning muscle fibers is directly correlated with muscle function that stabilizes and moves the vertebral column. Along with muscle atrophy, fatty infiltration of the skeletal muscle (myosteatosis) is also an essential frailty process [33, 34]. Therefore, we presume that PMF is an important factor in OVCFs similar to or more than CSA.

Sarcopenia is a generalized muscle disorder, including low muscle strength, muscle quantity, and physical performance, eventually leading to a reduced QoL [15]. Systemic muscle atrophy can influence the atrophy of localized spinal muscle mass and function, increasing the incidence of a spinal fracture [35, 36]. This is because core muscles stabilize the spinal segment. However, this hypothesis remains controversial. Furthermore, some studies reported that the psoas index and/or back muscle atrophy were not independent risk factors for spinal compression fracture [37, 38]. This discrepancy might be due to a simple error in measuring the CSA without considering a fatty change. We report that not only CSA but also PMF, or preferentially PMF over CSA, should be considered as an independent risk factor for OVCFs.

Fatty infiltration of the skeletal muscle has been recognized as an important component of aging and frailty. The cellular origins of fatty accumulation in the muscle arise through various pathways induced by factors, including muscle injury, increased endogenous glucocorticoid levels with age, and unloading through prolonged bedrest [39]. This reduces insulin sensitivity and anabolic metabolism in the skeletal muscle, thus impairing muscle function due to decreased contractility [39] A previous literature review found an association between osteoporosis and increased fat infiltration of back extensor muscle, which cause a decrease in balance, eventually leading to the risk of fractures [40].

Identifying the factors associated with OVCFs is essential for preventing further fractures. The T-score of BMD is a robust and quantitative predictive factor for osteoporotic fracture risk [41]. However, it should be considered that the T-score sometimes shows discordance with osteoporotic fracture risk. Degenerated lumbar spine showed uncertainty for BMD measurement for aging [42]. Measurement of bone density can be affected by calcifying structures around the spine, which are the anterior and posterior longitudinal ligaments, ligament flavum, aorta, and interspinous ligament [43, 44]. Therefore, the prediction of fracture risk through only BMD is a challenge concerning the degenerative spine, especially in those who cannot use hip BMD due to history of hip fracture or surgery, and attempts are being made to compensate for this problem with a new method called the trabecular bone score to measure the microstructure of bones [45]. In this study, the PMF in the P-BMD group significantly decreased than that in the control group. Back muscle quality had a significant impact on OVCFs independent of BMD. These results show the possibility of another new tool to predict OVCF in patients with limitations in measuring BMD. It also suggests that preserving muscle quality from a young age before physical activity is restricted, regardless of BMD, is important for preventing fractures in older age, although the effect may vary depending on the basic muscle quality of each individual.

This study had some limitations. First, we did not analyze the entire area of the back muscles. We only used the L4–5 and L5–S1 levels as surrogates of the back muscles to compare the control and fracture groups with various fracture sites. However, there was no significant difference in the PMF of the three levels (fracture level, L4–5, and L5–S1). In a previous study, back muscle degeneration was correlated with aging [46]. Combining the findings of our study and previous studies, it is thought that there are similarities rather than differences between each level of PMF in a person. Therefore, the analysis of the multifidus muscle in the lower lumbar region can represent the degree of systemic back muscle degeneration. Second, this study did not consider other confounding variables that could affect the risk of OVCF. This study was conducted in women only. Since the participants who visited a university hospital were targeted, a racial factor could not be ruled out. Because this study was designed as a retrospective cohort, each participant’s family history, physical fitness, and comorbidities could not be further investigated, so they could not be considered in this study. Furthermore, the treatment for osteoporosis or osteopenia was not sufficiently investigated and was not considered in the participants’ selection. Therefore, further studies considering these factors are needed in the future.

## Conclusions

Fatty infiltration of the back muscle significantly affects OVCFs in older patients, independent of BMD. Therefore, physicians should pay attention to the state of the back muscles in older patients to prevent compression fractures.

## Data Availability

The datasets used and/or analyzed during the current study are available from the corresponding author upon reasonable request.

## Abbreviations

ANOVA: analysis of variance
BMD: bone mineral density
BMI: body mass index
CI: confidence interval
CSA: cross-sectional area
MRI: magnetic resonance imaging
O-BMD: osteoporosis BMD
OR: odds ratio
OVCF: osteoporotic vertebral compression fracture
P-BMD: osteopenia BMD
PMF: percentage of muscle fibers in the multifidus muscle
QoL: quality of life
